# INTERAGENCY COLLABORATION TO SUPPORT CAREGIVERS IN RURAL COMMUNITIES

**DOI:** 10.1093/geroni/igae098.2218

**Published:** 2024-12-31

**Authors:** Jacqueline Telonidis, Christopher Hernandez, Randall Rupper, Jennifer Morgan, Katherine Supiano, Linda S Edelman, Kara Dassel

**Affiliations:** University of Utah, Salt Lake City, Utah, United States; University of Utah, Salt Lake City, Utah, United States; Salt Lake City VA Healthcare System, Salt Lake City, Utah, United States; Utah State University, Logan, Utah, United States; University of Utah, Salt Lake City, Utah, United States; College of Nursing, Salt Lake City, Utah, United States; University of Utah, Salt Lake Cty, Utah, United States

## Abstract

The Utah Geriatric Education Consortium (UGEC) is a Health Resources and Services Administration (HRSA) Geriatrics Workforce Enhancement Program committed to providing age- and dementia-friendly education to primary care and geriatrics workforces and supporting caregivers of people with dementia. We partnered with three rural, multi-county Area Agencies on Aging (AAA) to design and implement hybrid dementia caregiver conferences. Other partners included the Utah Chapter of the Alzheimer’s Association, Utah Department of Health and Human Services, VA Salt Lake City Healthcare System Geriatric Research Education and Clinical Center, and Utah Department of Veterans and Military Affairs. Conference topics included: UGEC educational programs and resources; local AAA community-based programs and resources; dementia, depression, and delirium screening; understanding/responding to dementia-related behaviors; caregiver grief; VA healthcare enrollment and caregiver support programs. The conferences averaged 70.33 (range 54-92) in-person attendees and 12 (range 3-21) virtual attendees. Attendees reported positive levels of satisfaction on a 5-point scale, including that the conferences were engaging (X = 4.55) and effective (X = 4.62), provided examples useful to their education (X = 4.59), met stated goals (X = 4.53), and were better/a lot better compared to similar programs (X = 4.40). In-person attendees reported a sense of community amongst caregivers, and appreciation for the topics related to grief and caregiver support. Attendees suggested adding a caregiver panel, and more local long-term care services and support vendors. In conclusion, rural caregiver conferences which include in-person and virtual modalities meet the educational and flexibility needs of rural caregivers in Utah.

